# The Role of Prosocial and Aggressive Popularity Norm Combinations in Prosocial and Aggressive Friendship Processes

**DOI:** 10.1007/s10964-019-01088-x

**Published:** 2019-08-12

**Authors:** Lydia Laninga-Wijnen, Christian Steglich, Zeena Harakeh, Wilma Vollebergh, René Veenstra, Jan Kornelis Dijkstra

**Affiliations:** 1 Utrecht University, Utrecht, The Netherlands; 2 University of Groningen, Groningen, The Netherlands; 3 Institute for Analytical Sociology, Linköping University, Linköping, Sweden; 4 TNO, Leiden, The Netherlands

**Keywords:** Popularity norm, Aggression, Prosocial behavior, Friendship selection, Friendship influence

## Abstract

Prior work has shown that popular peers can set a powerful norm for the valence and salience of aggression in adolescent classrooms, which enhances aggressive friendship processes (selection, maintenance, influence). It is unknown, however, whether popular peers also set a norm for prosocial behavior that can buffer against aggressive friendship processes and stimulate prosocial friendship processes. This study examined the role of prosocial and aggressive popularity norm combinations in prosocial and aggressive friendship processes. Three waves of peer-nominated data were collected in the first- and second year of secondary school (*N* = 1816 students; 81 classrooms; *M*_age_ = 13.06; 50.5% girl). Longitudinal social network analyses indicate that prosocial popularity norms have most power to affect both prosocial and aggressive friendship processes when aggressive popularity norms are non-present. In prosocial classrooms (low aggressive and high prosocial popularity norms), friendship maintenance based on prosocial behavior is enhanced, whereas aggressive friendship processes are largely mitigated. Instead, when aggressive popularity norms are equally strong as prosocial norms (mixed classrooms) or even stronger than prosocial norms (aggressive classrooms), aggression is more important for friendship processes than prosocial behavior. These findings show that the prosocial behavior of popular peers may only buffer against aggressive friendship processes and stimulate prosocial friendship processes if these popular peers (or other popular peers in the classroom) abstain from aggression.

## Introduction

Adolescents spend a large part of the day in their classroom, and the proliferation of prosocial and aggressive behavior in classrooms is vital to adolescents’ social-emotional and academic adjustment (Jones et al. 2010). Aggressive and prosocial behavior may proliferate through a dynamic interplay of peer selection, maintenance, and influence processes (Dishion and Tipsord 2011). Selection and maintenance refer to adolescents selecting and keeping friends; for instance based on similarity; as similarity enhances predictability, mutual understanding, and trust (similarity attraction hypothesis; Byrne 1971). In turn, adolescents may become similar to their friends via *influence* processes, due to social pressure or imitation. Following reputational salience hypothesis (Hartup 1996), these friendship processes would mainly occur for behaviors that are an important tool to improve one’s social reputation such as popularity. Indeed, adolescents increasingly strive for popularity (LaFontana and Cillessen 2010) and behaviors associated with achieving this goal become of high valence to them. The concept of “popularity norms” captures the within-classroom association between behavior—such as prosocial and aggressive behavior—and popularity (Henry et al. 2000). To date, only two studies examined popularity norms’ role in friendship processes. They showed that friendship selection and influence related to aggression (Laninga-Wijnen et al. 2017), and friendship influence on risk attitudes (Rambaran et al. 2013) were strongest in classrooms where these behaviors or attitudes were rewarded with popularity.

There are, however, two important gaps in our understanding on popularity norms’ role in friendship processes. First, the valuable work on antisocial popularity norms has not been accompanied by an equivalent exploration on domains that protect against this risk or promote positive development. Evolutionary-psychological theories (Ellis et al. 2016) state that when adolescents can achieve their goals (e.g., popularity) through prosocial behavior, they will attach less value to aggression; which would enhance prosocial - and mitigate aggressive friendship processes; however, this assumption has been empirically unexplored. Second, prior work only examined popularity norms and friendship processes in *one* behavioral domain (same-behavior processes); but reality is often more complex: multiple norms and friendship processes may both co-exist and interplay. This is particularly true for prosocial and aggressive behavior, which used to be defined as two distinct but also partly overlapping dimensions that both *co-occur* and *interplay* within individuals, relationships and contexts (Card et al. 2008; Hawley and Bower 2018; Pellegrini 2008). The *interplay* of these behaviors may occur through two processes. First, strong norms for one behavior may discourage friendship processes for the other behavior (e.g., prosocial norms diminish aggressive friendship processes); reflecting *cross-behavior norm processes*. Second, prosocial and aggressive behavior may interplay at the dyadic friendship level, through *cross-behavior friendship processes*. Adolescents displaying certain behaviors may select their friends based on the combination with another type of behavior, such as when highly prosocial adolescents select lowly aggressive peers as friends (cross-behavior friendship *selection*). Moreover, cross-behavior friendship *influence* occurs when certain behaviors of friends influence *other* behaviors in adolescents (Giletta et al. 2013), for instance when prosocial friends diminish adolescents’ aggression. Importantly, the extent to which these cross-behavior processes take place, is likely to depend on whether aggression and prosocial behavior can be considered as mutually exclusive; for instance, when one behavior is rewarded with popularity whereas the other is not. However, a previous study—on partly the same data as the current study—found that prosocial and aggressive popularity norms can also co-occur, indicating that in some classrooms prosocial and aggressive behaviors are not mutually exclusive as both behaviors have the function to gain popularity. This previous study distinguished three classroom types: mixed classrooms with high prosocial and high aggressive popularity norms, prosocial classrooms with high prosocial and very low aggressive popularity norms, and aggressive classrooms with high aggressive and relatively low prosocial norms (Laninga-Wijnen et al. 2018). The current study extends upon this prior work by examining the role of these popularity norm combinations (aggressive, prosocial and mixed) in aggressive and prosocial friendship processes.

## Prosocial Classrooms and Friendship Processes

In classrooms with high prosocial popularity norms and non-present aggressive popularity norms, prosocial behavior is highly valued and reputationally salient, whereas aggression is not (Hartup 1996). In such classrooms, prosocial and aggressive behaviors can be viewed as mutually exclusive as they do not co-exist at the norm level, which may elicit cross-behavior processes. The prosocial popularity norm may therefore not only enhance friendship selection, maintenance and influence related to prosocial behavior (same-behavior norm processes); but also discourage friendship selection, maintenance and influence related to aggression (cross-behavior *norm* processes). The prosocial popularity norm may also encourage cross-behavior *friendship* processes. Regarding cross-behavior *friendship selection*, highly prosocial youth may be attracted to lowly aggressive friends, as these peers may share similar values and principles (Brechwald and Prinstein 2011). At the same time, highly aggressive youth may be attracted to highly prosocial peers as friends, as affiliation with these highly prosocial friends can be an effective way to achieve popularity (Dijkstra et al. 2010). These aggressive adolescents may consider the goal of becoming popular more important than sharing the same values or principles (LaFontana and Cillessen 2010). Next, regarding cross-behavior *friendship influence*, highly prosocial youth may diminish adolescents’ aggression, whereas highly aggressive friends may not have the power to diminish adolescents’ prosocial behavior, as prosocial behavior is highly valued and rewarding in this context (Hartup 1996). So far, no study examined prosocial popularity norms’ role in these friendship processes.

## Aggressive Classrooms and Friendship Processes

Aggressive classrooms are characterized by high aggressive popularity norms and relatively low prosocial popularity norms (Laninga-Wijnen et al. 2018). In these classrooms, the aggressive popularity norm may strengthen friendship selection, maintenance, and influence related to aggression (same-behavior norm processes); and mitigate friendship selection, maintenance and influence based on prosocial behavior (cross-behavior norm processes). Regarding cross-behavior friendship selection, highly aggressive adolescents may select lowly prosocial peers as friends (Brechwald and Prinstein 2011), whereas highly prosocial adolescents may be attracted to highly aggressive peers (LaFontana and Cillessen 2010). Regarding cross-behavior friendship influence, aggressive friends are expected to mitigate adolescents’ prosocial behavior, whereas prosocial friends may not diminish adolescents’ aggression over time. Previous research found aggressive popularity norms to strengthen same-behavior friendship selection and influence (but not maintenance) regarding aggression (Laninga-Wijnen et al. 2017); However, that research considered neither the potential co-existence of prosocial popularity norms, nor cross-behavior processes.

## Mixed Classrooms and Friendship Processes

In mixed classrooms, both prosocial and aggressive behaviors are rewarded with popularity (Laninga-Wijnen et al. 2018). To date, it has not been investigated what happens in such contexts regarding friendship processes, and two alternate hypotheses can be delineated. First, based on the reputational salience hypothesis, it could be argued that in mixed classrooms, both prosocial and aggressive behaviors are valuable and attractive tools to achieve popularity (Hartup 1996). Therefore, in these classrooms prosocial and aggressive behaviors are not mutually exclusive, and cross-behavior (norm- and friendship) processes may *not* take place. As such, the prosocial popularity norm may only enhance prosocial friendship processes, and *not* diminish aggressive friendship processes; and the aggressive popularity norm may only enhance aggressive friendship processes, and not diminish prosocial friendship processes (e.g., *no* cross-behavior *norm* processes). With regard to cross-behavior friendship selection, highly aggressive adolescents may select highly prosocial peers as friends, and vice versa. In line with this reasoning, a previous study found that aggressive adolescents selected prosocial peers as friends when they were both high in popularity (Logis et al. 2013). Cross-behavior friendship influence may not occur either: both behaviors can flourish next to each other, as adolescents may not feel the need to—for example—diminish their aggression based on the prosocial behavior of their friends, given that both behaviors are valuable strategies to gain popularity (Hawley and Bower 2018). In sum, a first hypothesis would be that if prosocial and aggressive behaviors are both of high valence to adolescents, they may independently co-exist and *not* interplay through cross-behavior processes. Consequently, prosocial friendship processes may be equally strong in mixed classrooms and prosocial classrooms (both classroom types have equivalent prosocial norms), and aggressive friendship processes may be equally strong in aggressive classrooms as in mixed classrooms (equivalent aggressive norms), and cross-behavior friendship processes may be non-existent.

Second, an alternate hypothesis can be proposed. There are reasons to assume that aggressive popularity norms may dominate prosocial popularity norms in affecting friendship processes, even when these norms are equally present. Various reviews in the psychological literature on the role of negative events in relation to positive events (Baumeister et al. 2001; Rozin and Royzman 2001) suggest that negative events or entities have a greater power over positive ones, and underpin this statement with prior research on—among others—life events, close relationship outcomes, social network patterns, interpersonal interactions, and learning processes. Possibly due to innate predispositions and experiences, human beings may give greater weight to negative entities (bad emotions, bad parents, and bad feedback from peers) than to positive ones (Rozin and Royzman 2001). Specific to the current study, a recent experimental study found visual attention towards popular peers to be stronger after a negative prime than after a positive prime, indicating that popular adolescents’ negative behaviors drive the greater attention they receive from their peers (Lansu and Troop-Gordon 2017). When adolescents attend more to popular peers’ aggression than to popular peers’ prosocial behavior, aggressive popularity norms could more strongly affect friendship processes than prosocial popularity norms, even when both norms are equally present. Combining reputational salience hypothesis with this literature on “the power of negative events”, makes it most likely to find support for the second hypothesis that aggressive norms would dominate prosocial norms, and hence, that mixed classrooms are relatively similar to aggressive classrooms in terms of friendship processes.

## Current Study

This study sought to understand the role of classroom combinations of prosocial and aggressive popularity norms in friendship processes related to prosocial and aggressive behavior. It is expected that friendship selection, maintenance and influence related to prosocial behavior would be stronger in prosocial classrooms than in aggressive classrooms, whereas friendship selection, maintenance and influence related to aggression would be stronger in aggressive than in prosocial classrooms. In prosocial classrooms, highly aggressive youth would be attracted toward highly prosocial friends, but not vice versa; and prosocial friends would diminish adolescents’ aggression, and not vice versa. In aggressive classrooms, it is expected that these cross-behavior selection and influence processes would be exactly the other way around. With regard to mixed classrooms, it is expected that aggressive norms would affect friendship processes more strongly than prosocial norms—therefore, mixed classrooms would be more similar to aggressive than to prosocial classrooms. For same-behavior processes, this study examined selection, maintenance and influence processes; whereas for cross-behavior processes this study only focused on selection and influence. This was done to prevent convergence issues, which were more likely to emerge in these highly complex models if cross-behavior maintenance (and other associated) effects would be included.

## Methods

### Participants and Procedure

Data originated from the SNARE-project (Social Networks and Risk Behavior in Early Adolescence). All first-year and second-year students in two secondary schools in the Netherlands were approached to take part in the project (Cohort 1) at the beginning of the academic year 2011–2012. A second cohort of students entering first year in these secondary schools was asked to take part in the project the following academic year 2012–2013 (Cohort 2). Data were collected three times in one academic year, in the fall, winter and spring of 2011–2012 (Cohort 1) and 2012–2013 (Cohort 2). Before data-collection started, students received an information letter describing the goal of the study and offering the possibility to refrain from participation. Parents who did not wish their children to participate in the study were asked to indicate this and students were made aware that they could cease their participation at any time. The survey was completed in the classroom by computer, supervised by a researcher, using Bright Answer socio software (SNARE software 2011). The privacy and anonymity of the students were warranted, and the study was approved by the Internal Review Board (IRB) of Utrecht University (see also Franken et al. 2016; the project name is “Social Network Processes and Social Development of Children and Adolescents”).

Of the 1854 approached first- and second-year students, 2.0% declined to participate. The final sample comprised 1816 first- and second-year students from 81 classrooms (63% first-year students), with 917 (50.5%) girls, aged between 11 and 15 years (*M* = 13.05, SD = 0.71). Each class consisted 12–30 students (*M* = 22.42 participating students per class). Of the participants, 48.1% were attending lower-level education (i.e., preparatory secondary school for technical and vocational training), whereas 51.9% were enrolled in higher-level education (including preparatory secondary school for higher professional education and for university). Most respondents were native Dutch (80.9%)

Participants’ socioeconomic status was assessed based on the zip codes, using “status scores” of the Social Cultural Planning Office, The Netherlands (see Benson et al. 2015). These status scores were based on the percentage of habitants with lower incomes, the percentage of lowly educated habitants, average income of habitants within an area, and the percentage of unemployed habitants. It was not possible to define the social status of 9.7% of the sample, because these participants had not filled in their zip code or because the zip code was not in the system of the Social Cultural Planning Office. About a third of the participants (32.3%) came from areas with lower socioeconomic status, whereas 50.8% came from areas with an average socioeconomic status. The smallest percentage of participants (7.2%) came from areas with a higher socio-economic status.

### Measures

All research variables were based on peer nominations, measured at each of the three waves (T1, T2, and T3). Peer-nominated variables were assessed by asking participants questions about their classmates. Adolescents were told that they could nominate an unlimited number of same-gender and opposite-gender classmates. There was also the option of selecting “nobody”, so that it was possible to differentiate between missing responses and valid empty responses in the name generators. Names were presented in random order to avoid (alphabetical) answer tendencies.

### Friendship (Dyadic Measure)

Participants received a list of all consenting students in their class. They were asked to nominate their best friends within the classroom. Based on these nominations, an adjacency matrix was constructed, containing all within-classroom friendship nominations of all classrooms across the three waves.

### Aggressive Behavior (Individual-Level Attribute)

Peer-perceived aggressive behavior was assessed using within-classroom peer nominations on four items about aggressive behavior: “Who bullies you?”; “Who quarrels and/or initiates fights with you?”; “Who sometimes spreads rumors or gossips about you?”; and “Who makes fun of others?” (see also Hamre and Pianta 2006; Lease et al. 2002; Logis et al. 2013; Molano et al. 2013; Laninga-Wijnen et al. 2017). For each item, the number of received nominations was divided by the number of nominators, so that scores represented the proportion of classmates that had nominated an adolescent for that item. Principal component factor analyses for the three waves showed that these four items represented one factor, explaining 62.2–67.9% of the variance (factor loadings varying from 0.73 to 0.86). Therefore, these items were averaged for each wave to create a scale for aggressive behavior, which represented the average percentage of peers who nominated a particular adolescent as aggressive using the four items. Scores on this scale varied from 0 (=nominated by nobody on the four items) to 1 (nominated by everyone on all four items). Cronbach’s alphas were *α*_*T*1_ = 0.73, *α*_*T*2_ = 0.79 and *α*_*T*3_ = 0.76 respectively, indicating good internal consistency. Because RSiena analyses (Simulation Investigation for Empirical Network Analyses) require ordinal categorical dependent behavior variables, the peer-nominated aggressive behavior was recoded into four roughly equally populated categories based on quartiles of the variable’s distribution pooled over all classes and time points (in line with previous studies, Laninga-Wijnen et al. 2018).

### Prosocial Behavior (Individual-Level Attribute)

Peer-perceived prosocial behavior was assessed using peer nominations on three items: “Who gives others the feeling that they belong to the group?”; “Who helps others by giving good advice?”; and “Who help you with problems (e.g., with homework, repairing a flat tire, or when you feel down)?”), (see also Laninga-Wijnen et al. 2018). For each item, the number of received nominations was divided by the number of nominators, so that scores represented the proportion of classmates that had nominated an adolescent for that item. Principal component factor analyses for the three waves showed that these three items represented one factor, explaining 64.3–72.7% of the variance (factor loadings ranging from 0.77 to 0.88). For each wave, the average of these three items was used as a scale for prosocial behavior. Cronbach’s alphas of the resultant scale were *α*_*T*1_*=* 0.72, *α*_*T*2 = _0.75, and *α*_*T*3_ = 0.81, respectively, indicating sufficient and good internal consistency. In order to use this scale for RSiena analyses, peer-nominated prosocial behavior was recoded into four roughly equally populated categories based on quartiles of the variable’s distribution pooled over all classes and time points.

### Popularity Norms (Classroom-Level)

Popularity norms for aggression and prosocial behavior at T1 were calculated for each classroom as the correlation between peer-nominated aggressive or prosocial behavior and popularity, respectively (Dijkstra and Gest 2015; Dijkstra et al. 2008). Peer-nominated popularity was assessed by asking participants “Who is most popular?” and “Who is least popular?” (correlation between these items is *r=* −0.45). For each item, the number of received nominations was divided by the number of nominators, so that scores represented the proportion of classmates who had nominated an adolescent for that item. The score for least popular was subtracted from the score for most popular to obtain a single continuum of popularity (e.g., Lease et al. 2002; Cillessen and Rose 2005).

### Analytic Strategy

#### Attrition analyses

Percentages of participants with missing values were 1.6 % at wave 1, and 1.4% at both wave 2 and wave 3. Missing data analysis showed no significant or substantial differences between partially missing cases and complete cases across time points. Missing data due to nonresponse were handled using the SIENA missing data method (Huisman and Steglich 2008) with the “last observation carry forward” method proposed by Huisman and Snijders 2003 (LOCF; 2003).

#### Classroom popularity norm combinations

In order to identify different classroom combinations (or “profiles”) based on aggressive and prosocial popularity norms, iterative *k*-cluster analysis was conducted in SPSS (version 25). Cluster analysis allows at identifying relatively homogenous groups using information across multiple variables because its algorithm maximizes within-group homogeneity and does not require an arbitrary and complex set of a priori cut-scores. Based on a previous study on partly the same data as the current study (Laninga-Wijnen et al. 2018), a three-cluster solution was expected, but it was compared to a two-, four-, and five-cluster solution to test whether a three-cluster solution was indeed preferable based on the content of the profiles and minimal number of classrooms in a profile. In line with previous studies using *k*-cluster analysis (Dijkstra and Gest 2015) the following criteria were used to decide upon the cluster-solution: clusters should provide distinct new profiles and should contain at least 5% of the total sample of classrooms. Also, the distance table should indicate that both norms contribute to the cluster-solution.

#### Friendship processes

This study used longitudinal social network analyses (Snijders 2005) implemented using the Simulation Investigation for Empirical Network Analysis (RSienaTest) software package in R (RSienaTest version 1.1–352) to analyze friendship processes related to prosocial and aggressive behavior. The RSiena program estimates the extent to which similarity among friends (in aggression and prosocial behavior) is due to same-behavior and cross-behavior friendship selection, maintenance, and influence processes, while controlling for structural network effects and the overall development of aggressive and prosocial behavior in the network. In Appendix (A) the model specification of these control parameters is discussed.

In order to achieve high statistical power while sufficiently accounting for potential heterogeneity between classrooms with the same popularity norm combination, a random effects model with Bayesian estimation methods was used (see Section 12.3; Ripley et al. 2017). In short, Bayesian inference assigns a prior probability distribution to the parameter—which is, in the light of new data, updated to a posterior probability. The posterior probability density is proportional to the product of the prior density and the likelihood of the data. Computations are made using Markov Chain Monte Carlo algorithms (Koskinen and Snijders 2007; Ripley et al. 2017). All control variables were allowed to randomly vary between classrooms within the same popularity norm profile, whereas parameters corresponding to hypotheses were assumed to be constant in these classrooms in order to gain power (the null hypothesis is that they are 0, and therefore constant; see Ripley et al. 2017). Posterior means and standard deviations for the fixed parameters *η* and the random parameters *μ* will be reported, as well as variation between classrooms for the random parameters, indicated by *τ*^2^ and *sd (τ*^2^*)*.

##### Model specification friendship same-behavior selection processes

In order to examine the extent to which friendship selection related to aggression and prosocial behavior took place, several effects were estimated, both for prosocial and aggressive behavior. The “*effect of behavior on friendship nominations received”* indicates whether adolescents with high levels of aggressive or prosocial behavior are more often nominated as friends. Conversely, the "*effect of behavior on friendship nominations given"* indicates whether adolescents with high levels of aggressive or prosocial behavior have a higher tendency to *give* more friend nominations to peers. Moreover, the estimated squared functions of these estimates were included in the models (EgoSqX and AltSqX; Snijders and Lomi 2019). By including these effects, the parameter “*similarity-based selection”* (Ego*Alter creation^1^), for both prosocial and aggressive behavior, provides reliable estimates for testing hypothesis about the extent to which adolescents form new friendships with peers based on similarity in aggressive and prosocial behavior.

##### Model specification friendship same-behavior maintenance processes

It was examined to what extent being similar in aggressive or prosocial behavior would predict that a friendship present at one measurement is still present at the next measurement (using Ego*Alter endowment effects). A positive parameter for similarity-based maintenance of friends indicates that similarity in aggressive and prosocial behavior predicts friendship *maintenance*.

##### Model specification friendship same-behavior influence processes

The behavioral dynamics of the model consisted of several control effects, see Appendix (A). Same-behavior friendship influence was measured with the average alter parameter. This represents the tendency of adolescents to develop their behavior toward the values of their friends’ behavior; which can work in an upward or in a downward direction (or remain similar)—depending on how aggressive or prosocial adolescents’ friends are.

##### Model specification cross-behavior friendship processes

The interacting cross-behavior friendship selection effects between prosocial and aggressive behavior, such as the *prosocial ego* * a*ggression alter* effect were included. A negative parameter for cross-behavior selection implies that adolescents with high (low) scores one type of behavior, tend to select friends who score low (high) on the other type of behavior; for instance, that highly prosocial adolescents select lowly aggressive friends. Moreover, the cross-behavior friendship influence (avXAlt) parameter indicated whether a friends’ behavior in one domain, influenced adolescents’ behavior in another domain. A negative parameter for cross-behavior influence indicates that friends that are high (low) in one type of behavior influence adolescents toward lower (higher) levels of the other type of behavior; for instance, that highly prosocial friends diminish adolescents’ aggression over time. Both endowment (decrease) and evaluation (increase) effects were estimated, to more specifically examine the direction of cross-behavior influence effects.

##### The moderating role of popularity norm combinations

In order to test whether popularity norm combinations moderate friendship same-behavior and cross-behavior processes, analyses were first performed for *all* classrooms and next, for aggressive, mixed, and prosocial classrooms separately. Classrooms were compared with each other based on *p*-values and based on credibility intervals of estimates. The *p*-values indicate the posterior probability that the parameter is greater than 0, and the chance that the parameter is smaller than 0 can be retrieved by 1 − *p*. *P-*values of ≥0.95 and ≤.05 reflect a high posterior chance that the alternate hypothesis is true (≥95% in both scenarios). If certain estimates are highly likely in the one classroom type (*p*-values for estimates ≥0.95 or ≤0.05) but not in other classroom types (0.05 < *p<* 0.95), this indicates differences between classrooms. Moreover, credibility intervals represent the range of values for the parameter that has a posterior probability of 0.95; and these were used to compare estimates between classrooms. If credibility intervals for estimates of different classrooms did not overlap, these estimates were considered to differ from each other. If applicable, ego-alter tables were calculated to further examine between-classroom differences in the direction of selection, maintenance, and influence effects.

## Results

### Popularity Norm Combinations

The prosocial popularity norm varied from −0.14 to 0.93 across classrooms (*M* = 0.48; SD = 0.23; 95% range = 0.06–0.85) and the aggressive popularity norm varied from −0.52 to 0.81 across classrooms (*M=* 0.33; SD = 0.30; 95% range = −0.35–0.70). These correlations indicate that classrooms varied largely in both the prosocial popularity norm and the aggressive popularity norm. The correlation between prosocial and aggressive popularity norms was weakly negative (*r=* −0.22, *p=* 0.051).

**Fig. 1:**
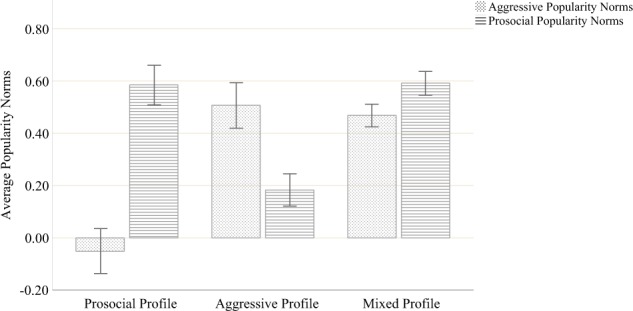
Three-cluster solution of popularity norm combinations (*N* = 81 classrooms) at wave 1

Based on iterative *K*-cluster analyses, a three-class solution was found to be superior, as this class solution rendered three meaningfully distinct configurations of prosocial and aggressive popularity norms with sufficient classrooms within each profile (Fig. 1). A four-class and a five-class solution did not provide distinct new profiles: extra profiles were variations based on a profile that was already present in the three-class solution, and the number of classrooms within additional profiles was rather low (<5% of the total sample of classrooms). The distance table of the *k* – cluster analysis indicates that both aggressive and prosocial popularity norm variations significantly contribute to the three-cluster solution (both *p<* 0.001).

Prosocial classrooms (*N* = 23) were characterized by high prosocial popularity norms and significantly lower (even negative) aggressive popularity norms, as computed with a dependent samples *t*-test [*t*(22) = −12.64, *p<* 0.001]. Aggressive classrooms (*N=* 22) were characterized by high aggressive popularity norms and significantly lower prosocial popularity norms [*t*(21) = 6.93, *p<* 0.001]. *Mixed* classrooms (*N=* 36) were characterized by high aggressive popularity norms and significantly higher prosocial popularity norms [*t*(35) = −4.26, *p<* 0.001]. Furthermore, comparing the three types of classrooms using ANOVA with Fisher’s least significant difference (LSD), aggressive popularity norms were found to be significantly lowest in prosocial classrooms (*p<* 0.001) but were equally high in aggressive and mixed classrooms (*p=* 0.40). Prosocial popularity norms were significantly lowest in aggressive classrooms (*p<* 0.001) but were equally high in prosocial and mixed classrooms (*p=* 0.87).

It was tested whether certain classroom types were represented more in first-year classrooms compared to in second-year classrooms, but this was not the case [χ^2^ (2) = 1.90, *p* = 0.39; *Φ* = 0.15]. Also, educational level was equally represented in the different classroom types [χ^2^ (2) = 1.45, *p* = 0.48; *Φ* = 0.13].

### Popularity Norm Combinations and Aggressive and Prosocial Friendship Processes

#### Descriptive results

Table 1 provides a description of friendships, prosocial behavior and aggressive behavior for the three classroom types. On average, youth nominated four to six classmates as their best friend. More than half of all friendships were reciprocated, and most friendships were between same-gender peers. Boys were perceived to display higher levels of aggressive behavior and girls higher levels of prosocial behavior. Jaccard Index was about 45%, indicating sufficient stability for social network analyses (Veenstra et al. 2013). Network autocorrelation indices (Moran’s *I*) were relatively high for prosocial and aggressive behavior, indicating that it is useful to conduct social network analyses to examine which processes (selection, maintenance or influence) underlie this autocorrelation. Moreover, prosocial classrooms were characterized by significantly more friendships and higher levels of prosocial behavior when compared to aggressive and mixed classrooms, consistently across time points; whereas levels of prosocial behavior were equal in mixed and aggressive classrooms. Also, aggressive classrooms scored consistently higher on aggression; these differences were significant when compared to mixed classrooms (T1 and T2) and prosocial classrooms (T2). This indicates that patterns of behavior and friendship vary between different types of classrooms.

**Table 1:** Description of the sample, network characteristics, and behavioral attributes for classrooms with aggressive, prosocial and mixed popularity norm combinations

	Prosocial popularity norm classrooms (*N=* 23)			Mixed popularity norm classrooms (*N=* 36)			Aggressive popularity norm classrooms (*N=* 22)		
	T1	T2	T3	T1	T2	T3	T1	T2	T3
	*M* (SD)	*M* (SD)	*M* (SD)	*M* (SD)	*M* (SD)	*M* (SD)	*M* (SD)	*M* (SD)	*M* (SD)
Network indicators									
Average number of friends	5.86 (3.06)^a^	6.13 (3.13)^a^	5.88 (2.86)^a^	5.06 (2.58)^b^	5.52 (2.69)^b^	5.25 (2.56)^b^	4.92 (2.43)^b^	5.52 (2.69)^b^	5.31 (2.82)^b^
Cohesion in friendship network	0.27 (0.08)	0.29 (0.08)	0.28 (0.07)	0.23 (0.06)	0.25 (0.05)	0.24 (0.06)	0.24 (0.08)	0.26 (0.06)	0.25 (0.08)
Proportion reciprocated relationships	61.7% (10.7%)	60.9% (10.3%)	63.4% (9.2%)	63.8% (9.4%)	64.3% (9.8%)	65.5% (12.5%)	67.2% (10.5%)	64.1% (9.7%)	62.1% (11.9%)
Proportion triadic friendships	64.3% (9.1%)	64.6% (8.3%)	65.2% (7.1%)	62.7% (9.7%)	64.6% (9.4%)	63.4% (11.5%)	61.8% (10.1%)	62.6% (8.6%)	63.1% (11.1%)
Proportion same-gender friendships	82.9% (9.9%)	80.2% (9.5%)	83.5% (8.6%)	85.9% (8.8%)	87.3% (9.4%)	86.8% (8.8%)	88.8% (8.5%)	88.6% (8.8%)	87.4% (9.9%)
Aggressive behavior									
Total average	0.03 (0.04)^ab^	0.04 (0.04)^a^	0.04 (0.05)^a^	0.03 (0.04)^b^	0.03 (0.04)^a^	0.04 (0.06)^a^	0.04 (0.06)^a^	0.04 (0.06)^b^	0.04 (0.06)^a^
Average boys	0.04 (0.05)^a^	0.04 (0.05)^a^	0.05 (0.05)^a^	0.06 (0.08)^a^	0.07 (0.08)^a^	0.07 (0.06)^a^	0.05 (0.07)^a^	0.06 (0.07)^a^	0.06 (0.07)^a^
Average girls	0.03 (0.03)^b^	0.03 (0.04)^b^	0.03 (0.04)^b^	0.03 (0.05)^a^	0.03 (0.06)^b^	0.03 (0.06)^b^	0.02 (0.04)^b^	0.03 (0.05)^b^	0.02 (0.04)^b^
Average highly popular students	0.05 (0.04)^b^	0.06 (0.06)^b^	0.06 (0.06)^b^	0.06 (0.05)^a^	0.07 (0.06)^a^	0.06 (0.09)^a^	0.07 (0.06)^a^	0.11 (0.09)^a^	0.10 (0.08)^a^
Average moderately popular students	0.03 (0.04)^a^	0.03 (0.04)^a^	0.04 (0.04)^a^	0.03 (0.04)^b^	0.03 (0.04)^b^	0.04 (0.05)^b^	0.03 (0.04)^b^	0.03 (0.05)^b^	0.04 (0.06)^b^
Average non-popular students	0.04 (0.05)^ab^	0.03 (0.05)^a^	0.03 (0.04)^a^	0.01 (0.02)^c^	0.02 (0.04)^c^	0.04 (0.02)^b^	0.03 (0.04)^b^	0.03 (0.06)^b^	0.02 (0.03)^b^
Prosocial behavior									
Total average	0.13 (0.07)^a^	0.13 (0.07)^a^	0.12 (0.08)^a^	0.11 (0.06)^b^	0.11 (0.06)^b^	0.11 (0.07)^b^	0.11 (0.07)^b^	0.11 (0.08)^b^	0.10 (0.07)^b^
Average boys	0.10 (0.05)^a^	0.11 (0.06)^a^	0.10 (0.07)^a^	0.08 (0.05)^a^	0.09 (0.06)^b^	0.08 (0.07)^a^	0.08 (0.05)^a^	0.09 (0.07)^a^	0.08 (0.06)^a^
Average girls	0.15 (0.08)^b^	0.15 (0.07)^b^	0.14 (0.09)^b^	0.11 (0.06)^a^	0.15 (0.08)^a^	0.13 (0.07)^b^	0.13 (0.06)^b^	0.14 (0.08)^b^	0.12 (0.08)^b^
Average highly popular students	0.17 (0.07)^c^	0.19 (0.08)^c^	0.18 (0.10)^a^	0.16 (0.06)^a^	0.14 (0.07)^a^	0.15 (0.08)^a^	0.15 (0.08)^a^	0.13 (0.08)^a^	0.13 (0.09)^a^
Average moderately popular students	0.13 (0.07)^b^	0.13 (0.06)^b^	0.11 (0.08)^b^	0.11 (0.06)^b^	0.11 (0.06)^b^	0.11 (0.07)^b^	0.11 (0.07)^b^	0.12 (0.08)^a^	0.10 (0.07)^b^
Average non-popular students	0.07 (0.05)^a^	0.07 (0.04)^a^	0.06 (0.05)^c^	0.06 (0.04)^c^	0.07 (0.05)^c^	0.07 (0.05)^c^	0.07 (0.05)^c^	0.06 (0.05)^b^	0.06 (0.06)^c^
Correlation prosocial behavior and aggression	−0.12*	−0.13*	−0.06	0.00	−0.09*	−0.03	−0.15***	−0.11*	−0.22***
Respondents									
% boys	49.8%^a^			50.2%^a^			51.6%^b^		
Probability similarity in friendship dyads in aggression (Moran’s *I*)	0.09	0.15	0.13	0.09	0.16	0.18	0.21	0.16	0.15
Probability similarity in friendship dyads in prosocial behavior (Moran’s *I*)	0.25	0.22	0.21	0.19	0.16	0.20	0.31	0.23	0.22
		T1–T2	T2–T3		T1–T2	T2–T3		T1–T2	T2–T3
Friendship change									
Average number of friendship changes		73.14	67.72		75.22	72.22		71.95	73.14
Proportion of stable friendships		0.55 (0.11)	0.53 (0.11)		0.50 (0.14)	0.51 (0.10)		0.51 (0.10)	0.53 (0.09)

Differences between (percentage of) boys and girls and different types of popular peers (non-popular, moderately popular and highly popular, based on +/− 1 SD relative to *M*) were calculated with ANOVA’s and indicated with superscripts letters

*p< 0.05; **p< 0.01; ***p< 0.001

#### Friendship processes in all classrooms

Table 2 displays the results of social network analyses performed on *all* classrooms, without considering popularity norms’ role, see Appendix (B) for control effects. Regarding friendship same-behavior processes, adolescents selected and maintained friendships with peers who are similar in aggressive and prosocial behavior (all *p>* 0.99; indicating that the posterior chance that these processes take place >99%). Moreover, adolescents were influenced by their friends’ aggressive and prosocial behavior (both *p>* 0.99).

**Table 2:** Longitudinal Bayesian social network analyses on friendship selection, maintenance and influence related to prosocial and aggressive behavior in all classrooms (*N* = 81 classrooms)

	Random			Fixed			Class-level variation	
*Network dynamics*	*μ*	sd (*μ*)	*p*	*η*	sd (*η*)	*p*	*τ*^*2*^	sd (*τ*^*2*^)
Friendship cohesion (density)	−0.91	0.17	<0.01				1.84	0.39
Reciprocity in friendship	1.61	0.07	>0.99				0.30	0.05
Transitive group formation (gwespFF)	1.64	0.06	>0.99				0.32	0.05
Transitive group formation (gwespBB)	0.23	0.06	>0.99				0.26	0.04
Indegree popularity (sqrt)	−0.55	0.06	<0.01				0.22	0.04
Outdegree popularity (sqrt)	−0.63	0.05	<0.01				0.21	0.03
Outdegree activity (sqrt)	0.00	0.04	0.50				0.17	0.02
Effect of gender on nominations received*	0.07	0.05	0.91				0.27	0.04
Effect of gender on nominations given*	−0.06	0.07	0.20				0.32	0.05
Same gender friendships	0.55	0.05	>0.99				0.31	0.05
Effect of prosocial behavior on nominations received				0.37	0.02	>0.99		
Squared effect of prosocial behavior on nominations received				−0.11	0.02	<0.01		
Effect of prosocial behavior on nominations given				−0.46	0.04	<0.01		
Squared effect of prosocial behavior on nominations given				0.09	0.04	>0.99		
Selection based on similarity in prosocial behavior				0.12	0.03	>0.99		
Maintenance based on similarity in prosocial behavior				0.15	0.04	>0.99		
Effect of aggression on friendship nominations received				−0.02	0.01	0.09		
Squared effect of aggression on friendship nominations received				−0.03	0.02	0.05		
Effect of aggression of friendship nominations given				−0.04	0.02	0.02		
Squared effect of aggression of friendship nominations given				−0.02	0.02	0.21		
Selection based on similarity in aggression				0.06	0.02	>0.99		
Maintenance based on similarity in aggression				0.27	0.03	>0.99		
Prosocial ego * aggressive alter: prosocial adolescents select aggressive friends				0.06	0.02	>0.99		
Aggressive ego * prosocial alter: aggressive adolescents select prosocial friends				0.02	0.02	0.82		
*Behavior dynamics*								
Prosocial behavior: linear shape	–2.39	0.17	<0.01				1.27	0.19
Prosocial behavior: quadratic shape	−0.48	0.05	<0.01				0.26	0.04
Prosocial behavior: indegree				0.35	0.02	>0.99		
Prosocial behavior: outdegree				0.08	0.01	>0.99		
Prosocial behavior: effect from gender	−0.94	0.11	<0.01				0.68	0.13
Prosocial behavior: effect from aggression				−0.08	0.04	0.02		
Influence prosocial friends on prosocial behavior adolescent				0.26	0.08	>0.99		
Influence aggressive friends on prosocial behavior adolescent (evaluation)				−0.32	0.17	0.01		
Influence aggressive friends on prosocial behavior adolescent (endowment)				0.12	0.30	0.63		
Aggressive behavior: linear shape	−0.07	0.15	0.31				0.68	0.12
Aggressive behavior: quadratic shape	0.03	0.05	0.72				0.35	0.04
Aggressive behavior: indegree				0.00	0.02	0.51		
Aggressive behavior: outdegree				−0.01	0.01	0.31		
Aggressive behavior: effect from gender	0.33	0.10	>0.99				0.52	0.10
Aggressive behavior: effect from prosocial behavior				−0.04	0.06	0.27		
Influence aggressive friends on adolescent’ aggression				0.47	0.07	>0.99		
Influence prosocial friends on aggression adolescent (evaluation)				0.32	0.15	0.99		
Influence prosocial friends on aggression adolescent (endowment)				−0.06	0.30	0.42		

Posterior means *η* and standard deviations sd (*η*) for fixed parameters. Posterior means *μ* and sd (*μ*) for random parameters. The *p* represents the percentile of zero in the posterior distribution. Asterisk indicates girls are reference category

Regarding cross-behavior friendship processes, there was a positive cross-behavior friendship selection effect for prosocial ego * aggressive alter. Together with the negative prosocial ego effect (−0.46) and negative aggressive alter effect (−0.02), this can be interpreted as lowly prosocial adolescents selecting lowly aggressive peers as friends. There was no aggressive ego * prosocial alter effect, indicating that aggressive adolescents were not more likely to select lowly (or highly) prosocial peers as friends. For cross-behavior friendship influence processes, it appeared that adolescents with relatively more aggressive friends were more likely to decrease in prosocial behavior, whereas adolescents with relatively fewer aggressive friends were more likely to increase in prosocial behavior (negative evaluation effect; *η=* −0.32, *p=* 0.01). Adolescents with relatively more prosocial friends were more likely to increase in aggression—or less likely to decrease in aggression—over time (positive evaluation effect, *η=* 0.32).

#### Friendship processes in classrooms with different popularity norm combinations

In the next step, the same model was tested for the three types of classrooms (prosocial, aggressive, and mixed; Table 3). Estimates with different superscripts differed significantly from each other, as credibility intervals did not overlap.

**Table 3:** The role of popularity norm combinations in the strength of friendship selection, maintenance and socialization related to prosocial and aggressive behavior within the classroom

	Prosocial popularity norm classrooms				Mixed popularity norm classrooms				Aggressive popularity norm classrooms			
Parameters	*η*	sd(*η*)	CI	*p*	*η*	sd(*η*)	CI	*p*	*η*	sd(*η*)	CI	*p*
Prosocial behavior												
Effect of prosocial behavior on nominations received	0.38	0.08	0.26 to 0.50	>0.99	0.36	0.04	0.29 to 0.43	>0.99	0.41	0.04	0.34 to 0.50	>0.99
Squared effect of prosocial behavior on nominations received	−0.09^a^	0.07	−0.20 to 0.02	0.14	−0.10^a^	0.02	−0.15 to −0.06	<0.01	−0.17^b^	0.04	−0.26 to −0.09	<0.01
Effect of prosocial behavior on nominations given	−0.65^a^	0.05	−0.73 to −0.55	<0.01	−0.38^b^	0.03	−0.44 to −0.33	<0.01	−0.36^b^	0.04	−0.43 to −0.26	<0.01
Squared effect of prosocial behavior on nominations given	0.20^a^	0.07	0.08 to 0.36	>0.99	0.14^a^	0.04	0.08 to 0.23	>0.99	−0.04^b^	0.05	−0.13 to −0.05	0.19
Selection based on similarity in prosocial behavior	0.15	0.09	0.02 to 0.29	0.98	0.06	0.06	−0.05 to 0.16	0.80	0.16	0.05	0.06 to 0.27	>0.99
Maintenance based on similarity in prosocial behavior	0.40^a^	0.13	0.20 to 0.63	>0.99	0.14^ab^	0.06	0.05 to 0.25	>0.99	0.10^b^	0.05	0.00 to 0.21	0.97
Prosocial ego * aggressive alter (prosocial adolescents select aggressive friends)	0.13^a^	0.03	0.07 to 0.19	>0.99	0.04^ab^	0.03	0.00 to 0.10	0.98	−0.01^b^	0.03	−0.06 to 0.04	0.41
Influence of friends’ prosocial behavior on adolescents’ prosocial behavior	0.07	0.15	−0.24 to 0.36	0.66	0.35	0.13	0.12 to 0.61	0.99	0.20	0.18	−0.13 to 0.61	0.88
Adolescent’ prosocial behavior: effect of aggression	−0.12	0.09	−0.29 to 0.03	0.09	−0.04	0.06	−0.16 to 0.08	0.25	−0.16	0.10	−0.32 to 0.04	0.06
Influence aggressive friends on adolescents’ prosocial behavior (eval)	−0.22	0.35	−1.09 to 0.28	0.28	−0.38	0.34	−1.13 to 0.23	0.11	−0.66	0.32	−1.41 to −0.16	<0.01
Influence aggressive friends on adolescents’ prosocial behavior (endow)	−0.25	0.61	−1.44 to 1.10	0.32	0.19	0.61	−0.96 to 1.39	0.63	0.96	0.58	−0.08 to 2.12	0.96
Aggression												
Effect of aggression on friendship nominations received	−0.04	0.04	−0.12 to 0.03	0.19	−0.02	0.02	−0.06 to 0.03	0.16	0.00	0.03	−0.06 to 0.05	0.48
Squared effect of aggression on friendship nominations received	0.00	0.03	−0.06 to 0.06	0.51	−0.02	0.02	−0.06 to 0.03	0.16	−0.03	0.03	−0.09 to 0.03	0.21
Effect of aggression of friendship nominations given	−0.03	0.04	−0.12 to 0.04	0.25	−0.04	0.03	−0.09 to 0.01	0.04	−0.07	0.03	−0.12 to −0.01	<0.01
Squared effect of aggression of friendship nominations given^*^/Superscript>	−0.20	–	–	–	-0.01	0.03	−0.06 to 0.04	0.35	0.11	0.04	0.04 to 0.19	>0.99
Selection based on similarity in aggression	0.03	0.04	−0.03 to 0.11	0.81	0.08	0.03	0.01 to 0.15	0.99	0.03	0.04	−0.03 to 0.10	0.71
Maintenance based on similarity in aggression	0.34	0.08	0.21 to 0.53	>0.99	0.30	0.04	0.22 to 0.39	>0.99	0.19	0.05	0.09 to 0.28	>0.99
Aggressive ego * prosocial alter: aggressive adolescents select prosocial friends	0.02	0.03	−0.06 to 0.07	0.78	0.04	0.03	−0.02 to 0.09	0.92	0.01	0.03	−0.05 to 0.07	0.62
Influence of friends’ aggression on adolescents’ aggression	−0.01	0.13	−0.27 to 0.25	0.44	0.62^b^	0.10	0.44 to 0.85	>0.99	0.66^b^	0.12	0.43 to 0.92	>0.99
Adolescent’ aggressive behavior: effect of prosocial behavior	−0.15	0.12	−0.45 to 0.04	0.09	0.20	0.07	0.07 to 0.32	>0.99	−0.20	0.17	−0.56 to 0.09	0.09
Influence prosocial friends on aggression adolescent (eval)	0.53	0.29	0.05 to 1.12	0.99	0.15	0.24	−0.28 to 0.63	0.71	0.31	0.48	−0.49 to 1.46	0.73
Influence prosocial friends on aggression adolescent (endow)	−0.34	0.49	−1.38 to 0.54	0.25	0.00	0.46	−0.92 to 0.81	0.50	0.14	0.88	−1.76 to 1.51	0.63

^*^Superscript>This effect had to be fixed in prosocial classrooms to reach convergence criteria. Results with the fixed effect were similar to results with non-fixed effect. Parameters with different superscripts differ significantly from each other

##### Prosocial classrooms

In prosocial classrooms, prosocial behavior was important for friendship selection and maintenance, but not for friendship influence (*η=* 0.07, *p=* 0.66). Regarding *cross-behavior norm processes* (e.g., the role of prosocial norms in aggressive friendship processes), aggression only played a marginal role in friendship selection via the maintenance effect (*η=* 0.34, *p>* 0.99). Table 4 indicates that friendships were more likely to be maintained if friends were similarly *low* in aggression than when friends were similarly *high* in aggression [*OR*(exp.(0.41 − 0.21)) = 1.22]. Furthermore, aggressive friendship influence did not take place (*η=−*0.01, *p=* 0.66). As hypothesized, this indicates that the prosocial popularity norm mitigates aggressive friendship processes.

**Table 4:** Ego-alter friendship maintenance based on aggression in prosocial, mixed and aggressive classrooms

	Prosocial classrooms				Mixed classrooms				Aggressive classrooms			
	Friends’ aggression				Friends’ aggression				Friends’ aggression			
Adolescents’ aggression	1	2	3	4	1	2	3	4	1	2	3	4
1	0.41	−0.13	−0.67	−1.21	0.62	0.22	−0.23	−0.73	0.68	0.46	0.17	−0.17
2	0.27	0.07	−0.14	−0.34	0.17	0.07	−0.08	−0.27	0.12	0.09	0.00	−0.15
3	−0.27	−0.13	0.00	0.14	−0.30	−0.10	0.06	0.17	−0.22	−0.06	0.05	0.09
4	−0.12	−0.73	−0.26	0.21	−0.79	−0.28	0.18	0.59	−0.33	0.02	0.32	0.56

Numbers in the table reflect the strength of attraction for students to become friends with certain peers, given their own and their peers’ aggression levels (columns dependent on rows). The values in the cells in these tables can be transformed to odds by taking the exponential function (exp.[*k*])

Next, the prosocial norm affected *cross-behavior friendship processes*: adolescents who were low in prosocial behavior tended to select lowly aggressive adolescents as friends, reflected by the negative prosocial ego * negative aggressive alter effect (*η=* 0.13, *p>* 0.99). Next, aggressive friends did not diminish adolescents’ prosocial behavior. Instead, prosocial friends *did* affect adolescents’ aggression (*η=* 0.53, *p=* 0.99), but in a somewhat unexpected way. Adolescents were more likely to increase in aggression (or less likely to decrease in aggression) if they had highly prosocial friends, which was in contrast to our hypothesis. With this latter finding as exception, most findings in these prosocial classrooms indicate that prosocial norms enhance certain prosocial friendship processes and mitigate aggressive friendship processes.

##### Aggressive classrooms

In *aggressive classrooms*, aggression played a marginal role in friendship selection processes, with the maintenance effect as one exception (Table 4, column on the right). The influence effect indicated that respondents adjust their behavior to their friends’ aggression (*η=* 0.66, *p>* 0.99). Table 5 (column on the left) indicates that when adolescents change their aggression levels, they most strongly prefer their friends’ aggression levels when these are at the extreme ends of aggression (e.g., in *high* or in *low* aggression), which is indicated by having the largest differences between values within the upper row (1.63 to −2.03) and within the lowest row (−1.25 and 1.10). Moreover, regarding *cross-behavior norm processes* (role of aggressive norm in prosocial friendship processes): although prosocial behavior was important for friendship selection and maintenance (*η=* 0.10 and *η=* 0.16), it was less important when compared to prosocial classrooms, as indicated by non-overlapping credibility intervals. Moreover, prosocial friendship influence did not occur.

**Table 5:** Ego-alter influence table related to aggression in mixed and aggressive classrooms

	Aggressive classrooms				Mixed classrooms			
	Adolescent’ aggression				Adolescent’ aggression			
Friends’ aggression	1	2	3	4	1	2	3	4
1	1.63	0.52	−0.70	−2.03	1.02	0.30	−0.46	−1.27
2	0.67	0.23	−0.32	−0.99	0.15	0.05	−0.09	−0.28
3	−0.30	−0.06	0.05	0.05	−0.72	−0.20	0.28	0.71
4	−1.25	−0.36	0.43	1.10	−1.59	−0.45	0.65	1.70

Numbers in the table reflect the strength of peer influence on certain levels of aggression for the student resulting from the average levels of their best friends’ aggression (columns dependent on rows)

With regard to *cross-behavior friendship processes*, it appeared that aggressive friends diminished adolescents’ prosocial behavior (*η*_evaluation_*=* −0.66, *p<* 0.01; *η*_endowment = _0.96, *p=* 0.96), but not vice versa. In sum, as expected, in these aggressive classrooms, aggressive friendship processes are strongly present, whereas prosocial friendship processes are diminished.

##### Mixed classrooms

In mixed classrooms, aggressive behaviors were important for friendship selection and maintenance processes. Friendship influence on aggression was significantly stronger when compared to prosocial classrooms, and equal to aggressive classrooms (Table 3). The ego-alter table indicates that friendship influence on aggression again was most likely towards the extreme values of aggression, in particular *high* aggression, as differences between values within the lowest row were highest (−1.59 to 1.70; Table 5, column on the right). Prosocial behavior was not important for similarity-based selection, and only modestly for friendship maintenance. Friendship influence on prosocial behavior was significant in mixed classrooms. The ego-alter table indicates that this influence is most likely towards lower levels of prosocial behavior irrespective of friends’ prosocial behavior (indicated by the highest scores per row being in the first column; Table 6). These findings are largely in line with the hypothesis that aggressive popularity norms dominate prosocial popularity norms, as aggressive friendship processes are strongly present whereas prosocial friendship processes are mitigated (comparable to aggressive classrooms).

**Table 6:** Ego-alter influence table related to prosocial behavior in mixed classrooms

	Mixed classrooms			
	Adolescent’ prosocial behavior			
Friends’ prosocial behavior	1	2	3	4
1	2.69	1.09	−1.66	−5.55
2	2.19	0.94	−1.47	−5.02
3	1.69	0.78	−1.28	−4.48
4	1.19	0.63	−1.09	−3.94

Numbers in the table reflect the strength of peer influence on certain levels of prosocial behavior for the student resulting from the average levels of their best friends’ prosocial behavior (columns dependent on rows)

With regard to cross-behavior friendship processes, no cross-behavior selection or influence took place (except one small cross-behavior selection effect: lowly prosocial adolescents preferred lowly aggressive peers as friends). These findings are in line with the alternate hypothesis that both behaviors are considered as valuable due to their associations with popularity and therefore these behaviors are not mutually exclusive.

#### Sensitivity analysis

Several sensitivity analyses were run to check the robustness of findings. First, even though the three-cluster solution of the *k*-cluster analysis supports findings of a previous study (Laninga-Wijnen et al. 2018) that used Latent Cluster Analyses to identify clusters, it was examined whether a similar cluster solution would emerge when using another statistical approach: A two-step cluster analysis. In this analysis, the number of clusters was not fixed, in order to examine what cluster-solution would be detected in the data. Based on AIC (Burnham and Anderson 2002) and the log-likelihood criterium for determining distances between clusters, a three-factor solution again was detected. The cluster quality was indicated as good in the silhouette measure of cohesion and separation. The three-factor solution rendered by the two-step cluster analysis was almost identical to the cluster-solution rendered by the *k*-cluster analysis. Only *three* classrooms that were considered as mixed in the *k*-cluster analysis, were considered as prosocial in the two-step cluster analysis. Nevertheless, this implies that 96.3% of the classrooms were clustered in exactly similar ways, showing that the finding on classroom profiles is robust.

Next, sensitivity analyses were run to ensure that the social network analyses were robust to some changes in the variables and model. First, prosocial and aggressive behavior were coded into five rather than four roughly equally populated categories based on quintiles of the variable’s distribution pooled over all classes and time points, and all analyses (on all classrooms, and on prosocial, aggressive and mixed classrooms) produced highly comparable results, indicating that type of categorization did not affect the findings. Also, additional social network analyses were run without ego squared and alter squared effects, and without cross-behavior effects, to check whether the family of effects may not affect the findings, and results were the same.

## Discussion

Ushered in with pubertal and social changes, adolescents increasingly attach value to achieving popularity among their peers. Behaviors associated with popularity therefore become highly valuable and salient and may be used as a tool to increase adolescents’ popularity directly, or via affiliation with popular peers (Rambaran et al. 2013). As such, popular peers are assumed to set a norm (‘popularity norm’) for which behaviors are attractive and important in a particular context (Dijkstra and Gest 2015) and function as role models. Prior work found that in classrooms with strong aggressive popularity norms, adolescents prefer highly aggressive peers as friends and adopt their friends’ aggression, which may enhance the proliferation of aggression (Laninga-Wijnen et al. 2017). Unfortunately, a potential protective role of popular peers on more positive (such as prosocial) behaviors has not been considered to date. Moreover, aggressive and prosocial norms and friendship processes may both co-exist and interplay. For instance, one previous study—on partly the same data as the current study—used Latent Class Analysis to distinguish three classroom types: mixed (high prosocial and high aggressive popularity norms), prosocial (high prosocial and very low aggressive popularity norms), and aggressive classrooms (high aggressive and relatively low prosocial popularity norms; Laninga-Wijnen et al. 2018). The current study examined the role of these popularity norm combinations in prosocial and aggressive friendship processes. To this end, previously found classroom profiles (prosocial, aggressive, and mixed) were validated using another statistical approach (*k*-cluster analysis) and social network analyses were applied to examine how these profiles affect prosocial and aggressive friendship processes. Findings indicate that prosocial popularity norms encourage prosocial friendship processes and dampen aggressive friendship processes, but only when aggressive popularity norms are non-present or even negative (e.g., in prosocial classes). In contrast, aggressive popularity norms do have the power to diminish prosocial friendship processes and strongly encourage aggressive friendship processes, even in the presence of equally high prosocial popularity norms (e.g., in aggressive and mixed classes). In other words, a prosocial popularity norm is not able to buffer the impact of the aggressive popularity norm, while in contrast the aggressive popularity norm does buffer the impact of the prosocial popularity norm.

### Norm Combinations and Friendship Processes

#### Prosocial classrooms

In line with the reputational salience hypothesis (Hartup 1996), and evolutionary-psychological theory (Ellis et al. 2016), it was found that if prosocial rather than aggressive behaviors are an important tool to reach adolescents’ goal of popularity (e.g., in *prosocial classrooms)*, conditions for the proliferation of aggression are diminished, whereas conditions for the proliferation of prosocial behavior are enhanced. More specifically, in prosocial classrooms, adolescents did not choose their friends based on aggression, but based on high prosocial behavior; whereas friendships were most likely to be maintained if friends were *low* in aggression or high in prosocial behavior. Adolescents who were low in prosocial behavior did not choose highly aggressive adolescents as friends; instead, they selected lowly aggressive peers as friends. This can be considered as protective, as previous research suggests that lowly prosocial adolescents usually are more prone to engage in aggression to compensate for their lack of prosocial skills (Pepler et al. 2008; Obsuth et al. 2015), and having lowly aggressive friends makes this less likely. Moreover, prosocial norms buffered against the general tendency to adopt low prosocial behavior from lowly prosocial friends. Prosocial norms also diminished the influential role of aggressive friends: Aggressive friends did not have the power to enhance adolescents’ aggression or to decrease adolescents’ prosocial behavior in prosocial classrooms. In general, these findings illustrate that in prosocial classrooms, the prosocial behavior of popular peers may play a protective role by discouraging aggression (cross-behavior norm processes) and enhancing the importance of prosocial behavior.

There was one unexpected finding in prosocial classrooms: adolescents were more likely to increase in aggression—or less likely to decrease in aggression—if they had highly prosocial friends. This seems counter-intuitive, but can be explained in at least three ways. First, it could be that highly prosocial youth are more tolerant toward their friends and may be less inclined to speak up when their friends show aggression; because they do not want to get involved into fights with their friends themselves (Molano et al. 2013). In other words, highly prosocial friends may not put a strong brake on youth’ aggression. Therefore, aggressive youth with more prosocial friends are more likely to increase—or less likely to decrease—in aggression. Second, this effect may be induced by bi-strategic friends, referring to friends who are both high in prosocial and aggressive behaviors (Hawley 2003). It could be that in prosocial classrooms, only bi-strategic friends have the power to increase adolescents’ aggression. In line with this reasoning, a previous study found that prosocial adolescents cannot mitigate the role of bi-strategic adolescents in making aggression salient (Laninga-Wijnen et al. 2019). Third, there may be a statistical reason: aggression in prosocial classrooms is *so low*, hence the only way adolescents may change in this behavior, is by going up (regression to the mean).

#### Aggressive classrooms

In *aggressive classrooms*, there are both prosocial and aggressive norms, but aggressive popularity norms are significantly higher than prosocial popularity norms. Findings indicate that in such a situation, aggressive popularity norms overrule prosocial popularity norms in affecting friendship processes. Adolescents maintain their friends based on similarity in aggression, and friendship maintenance based on prosocial behavior is less important in aggressive classrooms than in prosocial classrooms. Prosocial friends do not have the power to change adolescents’ aggression or prosocial behavior, whereas aggressive friends have the power to diminish adolescents’ prosocial behavior and to enhance adolescents’ aggression.

#### Mixed classrooms

For *mixed classrooms*, most evidence was found for the hypothesis that aggressive popularity norms are stronger than prosocial popularity norms. Friends selected each other based on similarity in aggression and not on similarity in prosocial behavior. Moreover, friendship influence on aggression in mixed classrooms was similar to aggressive classrooms; and friends influenced each other towards lower prosocial behavior. Aggression may be inherently more overruling, visible, dominating and impactful behavior than prosocial behavior, which has been suggested by various studies reviewing psychological literature on the power of negative events over positive events (Baumeister et al. 2001; Rozin and Royzman 2001). As a result of innate predispositions and prior experiences, people seem to give greater weight to negative entities (negative behaviors, negative peer feedback) than to positive ones. Also, an experimental study found adolescents’ visual attention toward popular peers to be stronger after a negative prime than after a positive prime, indicating that the negative behaviors rather than the prosocial behaviors of popular peers drive the greater attention they receive from adolescents (Lansu and Troop-Gordon 2017). Because attention is a prerequisite for influence, adolescents may be more strongly influenced by aggressive popularity norms than by prosocial popularity norms. The finding that cross-behavior friendship processes were non-present supported the alternate hypothesis that in mixed classrooms, both behaviors are viewed as adaptive for reaching the goal of popularity and hence are not mutually exclusive (Hawley and Bower 2018). Consequently, adolescents do not feel the need to change their prosocial behavior in response to their friends’ aggression (or vice versa).

#### Strengths, limitations and future studies

Some limitations of the present study need to be acknowledged. First, popularity norms may change across the school year (Laninga-Wijnen et al. 2018). In the current study, most classrooms (about 70%) remained stable within the same popularity norm combination across the school year; however, some classrooms made a transition toward another popularity norm combination, mostly from an aggressive or prosocial type toward a mixed type. Due to power issues it was not possible to investigate whether these transitions also affected friendship processes related to aggressive and prosocial behavior over time. Future researchers are encouraged to collect larger samples and a higher number of classrooms and schools to examine potential trajectories in popularity norm combinations and its impact on friendship processes related to aggressive and prosocial behavior across the school year. This also enables researchers to examine whether the salience of aggression or prosocial behavior within classrooms depends on norms in a wider ecological level, such as the school (Bronfenbrenner 1979).

Second, in this study, peer-reported aggression was examined as a unified construct, without consideration for its different forms (physical vs. relational) and functions (reactive vs. proactive). Most items assessed relational forms of aggression. Also, one item assessed aggression against *others*, whereas the other three items were about aggression directed against the nominator. Given the nature of these latter questions, it could be that some aggressive students would not end up being nominated, even if they engaged in aggressive behaviors. However, previous studies have shown that youth generally tend to overestimate their peers’ antisocial behavior, such as aggression or deviant behavior (Prinstein and Wang 2005); particularly the antisocial behaviors of popular peers (Helms et al. 2014). Moreover, many aggressive acts such as bullying occur in private, and thus may be hidden from peers (e.g., see Olweus 2013). Therefore, the current study’s way of framing aggression items potentially mitigates adolescents’ general tendency to over-report on aggression. Moreover, all aggression items loaded strongly on one factor, the scale that was created was reliable across all waves, and deletion of the “who makes fun of others” item would result in a less desirable Cronbach’s alpha. Therefore, this measure of aggression is expected to adequately capture aggression in the classroom context, and future studies are encouraged to more narrowly compare adolescents’ reporting tendencies on individualized and general peer nomination items.

Third, it was not possible to examine to which extent students combined prosocial and aggressive behavior (bi-strategics; Hawley 2003) and how this affects friendship processes. For instance, the unexpected effect that aggressive adolescents are less likely to decrease (or more likely to increase) in aggression when they have more prosocial friends, could be due to the fact that these prosocial friends are also high on aggression (e.g., bi-strategics, Hawley and Bower 2018). Unfortunately, in the current study it is not possible to examine this properly, due to the complexity of already included effects. For instance, with regard to cross-behavior friendship influence, several avXAlt effects were included, which capture three-way interactions. An example of one included three-way interaction, is the interaction between (1) presence of friendship, (2) prosocial behavior of friend and (3) aggression of adolescent. Adding the aggression of a friend as the fourth term to be included in this interaction, would be too demanding for the model, and highly complex to interpret. Nevertheless, because this study is one of the first to examine cross-behavior influence effects and this is a highly complex and unexplored area, the current study provides an important first step in the literature.

The limitations notwithstanding, the current study has several strengths. First, whereas previous studies mainly examined popular peers as risk factor (Dijkstra et al. 2008; Laninga-Wijnen et al. 2017; Rambaran et al. 2013), this study illuminated the protective role of popular peers. It was shown that in prosocial classrooms, prosocial popular peers may buffer against aggressive friendship processes or encourage prosocial friendship maintenance. Future studies may examine how prosocial popularity norms can enhance friendship influence toward high prosocial behavior, rather than mitigate friendship influence toward low prosocial behavior; or examine why adolescents may increase in aggression in the presence of prosocial friends, despite high prosocial popularity norms. Second, this study shows the benefit of examining *combinations* of popularity norms—instead of only one popularity norm, as findings demonstrate that aggressive norms may dominate prosocial norms, even when both are equally present. Further research is encouraged to provide more insight in what contributes to the emergence of mixed classrooms and what factors may lead to multiple, somewhat contrasting norms co-existing within a setting. Third, this study is the first to examine *cross-behavior norm- and friendship processes*. In this way, this study made a next step in capturing reality’s complexity by acknowledging that the influence of norms and friendship is not bound to one behavioral domain; but that multiple behaviors interplay via cross-behavior norm- and friendship processes. Fourth, highly complex stochastic actor-based analyses were used, while taking into account the multi-level structure of the data. Moreover, the current study adopted the “five-factor model” that has been recently referred to as superior to more traditional methods for estimating selection processes (Snijders and Lomi 2019). Importantly, it was not possible to estimate endowment and creation functions for the ego and alter (squared) effects due to power issues, and therefore the findings for cross-behavior friendship selection should be interpreted with caution. Moreover, due to convergence issues, cross-behavior selection and maintenance processes could not be disentangled, which is an exciting avenue for future research.

## Conclusion

In adolescence, popular peers are highly visible and powerful, and function as role models by setting a norm (“popularity norm”) for which behaviors are attractive in a particular context (Dijkstra and Gest 2015). Accordingly, aggressive popularity norms have been shown to strengthen the selection of aggressive peers as friends, and adolescents’ tendency to adopt their friends’ aggression (Laninga-Wijnen et al. 2017). Unfortunately, the potential protective role of prosocial behaviors of popular peers has not been considered to date, and research did not consider the co-existence and interplay of multiple norms and friendship processes. The current study examined how aggressive and prosocial popularity norm combinations within classrooms relate to prosocial and aggressive friendship processes using social network analyses. Findings demonstrate that popular peers can be prosocial role models, as long as they (or other popular peers in their classroom) do not engage in aggression. More specifically, when only prosocial behaviors are reputationally salient, prosocial behavior may flourish via prosocial friendship processes, whereas the proliferation of aggression may be largely mitigated. Instead, aggressive popularity norms diminish prosocial friendship processes and enhance aggressive friendship processes, even in the presence of (equally high) prosocial popularity norms (e.g., in aggressive and mixed classrooms). Thus, this study shows that popular peers are powerful role models in adolescence by setting the norm for (the co-evolution of) peer relationships and behavioral development in the classrooms, and that popular peers’ aggressive behaviors have a stronger impact than their prosocial behaviors. It could be that in adolescence, aggression may be a stronger way to get attention from others, as it is a means to bridge the “maturity gap” and to stand up against adult-like values (Moffitt 1993). Theoretically, the findings of this study provide three key insights. First, the reputational salience of one behavior may affect friendship processes in another, related behavioral domain. Second, the reputational salience of a certain behavior can only be understood in relation to the reputational salience of other behaviors in the classroom. When multiple behaviors are equally rewarded with popularity (such as in mixed classrooms), it could still be that one norm dominates the other. Third, the reputational salience of a behavior may not only inform same-behavior friendship processes (Hartup 1996), but also cross-behavior friendship dynamics. Hence, this study informs—among others—reputational salience hypothesis on the importance of the co-existence and interplay of (reputationally salient) behaviors in adolescence. With regard to practical implications, interventions aiming at preventing or reducing aggressive (bullying) norms (such as the Meaningful Roles Intervention; Ellis et al. 2016) or at strengthening social-emotional core competences (SEL-programs, see Durlak et al. 2011) may need to not only encourage prosocial behavior by rewarding it with status, but to also actively discourage aggressive popularity norms. In this way, popular peers may be effective targets for promoting prosocial behavior and positive adjustment among youth within classrooms.

## Appendix (A)

### Model specification control parameters

In order to accurately assess friendship selection and maintenance processes, it is important to control for general network tendencies in the model. Therefore, the following control effects were included: *Rate parameters* refer to the rate of change in friendships between time points, indicating whether there is enough change in the friendship network (not reported for parsimony; available upon request). *Density* reflects the general tendency of select others as best friends. *Reciprocity* reflects the tendency to reciprocate received “best friend” nominations. *Transitive group formation* is measured with two “gwesp-effects”; that measure the tendency that “friends of friends become friends”. The *indegree-popularity* effect holds that adolescents who receive many nominations tend to receive more nominations over time, whereas the *outdegree-activity* effect reflects the tendency that adolescents who give many nominations will give more nominations over time. Finally, the *outdegree-popularity* effect represents adolescents who give many nominations to receive more nominations over time, and thus accounts for the relation between receiving and giving nominations. The inclusion of these effects accounts for observed degree differences in the data and offers protects against omitted variable bias related to ego- and alter effects of individual-level variables not included in the models (Ripley et al. 2017).

In order to accurately assess friendship influence on aggressive and prosocial behavior, this study controlled for the overall mean and variance of prosocial and aggressive behaviors by including the *linear shape* effect and the *quadratic shape* effect. For the latter effect, a negative parameter indicates pulling toward the mean, whereas a positive parameter indicates pushing away from the mean. Also, the effect of indegree on the behaviors was estimated (e.g., do received friendship nominations predict higher prosocial or aggressive behaviors?) as well as the outdegree (e.g., do adolescents with more given friendship nominations have a stronger tendency toward high values on prosocial or aggressive behavior?). Finally, this study controlled for the effect of gender, aggression and prosocial behavior (depending on the outcome variable) on behavioral tendencies in the network.

## Appendix (B)

### Control parameters

As control parameters were fairly similar in all models, only those of the first model on *all* classrooms will be interpreted (Table 2). A negative parameter for density (*η**=* −0.91; *OR* = 0.40) indicated that participants did not select everyone as a best friend. Adolescents reciprocated friendships (*η**=* 1.61; *OR* = 5.00) and were likely to become friends with the friends of their friends via forward (*η**=* 1.64; *OR* = 5.16) and backward (*η**=* 0.23; *OR* = 1.26) nominations. Adolescents who received many nominations received less nominations over time (*η**=* −0.55; *OR* = 0.58) and adolescents who gave many nominations decreased in nominations given over time (*η**=* −0.63; *OR* = 0.53). Also, adolescents were likely to select same-gender peers as friends (*η**=* 0.55; *OR* = 1.73). In general, boys were less prosocial and more aggressive compared to girls. General levels of prosocial behavior in the classroom were negatively affected by levels of aggression (*η**=* −0.08; *OR* = 0.91) whereas prosocial behavior did not affect levels of aggression (*η**=* −0.04; *OR* = 0.96).

## Footnotes

1 This effect measures a positive correlation between the behaviors of ego (adolescent) and alter (friend); hence it does not necessarily imply that ego and alter are behaving in exact similar ways.
